# Associations of body composition measures with circulating insulin-like growth factor-I, testosterone, and sex hormone-binding globulin concentrations in 16,000 men

**DOI:** 10.1038/s41366-024-01633-0

**Published:** 2024-10-21

**Authors:** Matthew C. Hynes, Cody Z. Watling, Yashvee Dunneram, Timothy J. Key, Aurora Perez-Cornago

**Affiliations:** Cancer Epidemiology Unit, Nuffield Department of Population Health, University of Oxford, Oxford, UK

**Keywords:** Obesity, Risk factors

## Abstract

**Background:**

Adiposity is positively associated with risk of some cancer sites and other health conditions in men; however, it is unclear if endogenous hormones play a role in these associations. We examined how body composition, measured from magnetic resonance imaging (MRI) and common measures of adiposity (e.g., body mass index (BMI)), are related to hormone concentrations in men from the UK Biobank study.

**Methods:**

Up to 16,237 men with available body composition data (including visceral, subcutaneous, and liver fat, muscle fat infiltration (MFI), lean tissue, and common adiposity measures) and serum hormone measurements (insulin-like growth factor-I (IGF-I), total testosterone, sex hormone-binding globulin (SHBG), and calculated free testosterone) were included. Multivariable-adjusted linear regression models were used to determine the geometric mean hormone and SHBG concentrations across categories of each exposure.

**Results:**

Common measurements of adiposity were highly correlated with MRI measures of central and total adiposity (*r* = 0.76–0.91), although correlations with ectopic fat (liver fat and MFI) were lower (*r* = 0.43–0.54). Most adiposity measurements showed an inverse U- or J-shaped association with circulating IGF-I and free testosterone; however, MFI was linearly inversely associated, and lean tissue volume was positively associated with both IGF-I and free testosterone concentrations. All body composition measures were inversely associated with total testosterone and SHBG concentrations (relative geometric mean difference between Q5 vs. Q1: 20–30%).

**Conclusion:**

Our results show that common adiposity and most MRI measures of adiposity relate similarly to serum hormone concentrations; however, associations with ectopic fat (particularly MFI) and lean tissue were different.

## Introduction

Excess body weight and obesity have been linked to a number of diseases in men, including hormone-related aggressive prostate cancer, colorectal cancer [1–3], and cardiovascular disease [4]. The potential mechanisms underlying these associations remain unclear, and may be related to the endocrine, paracrine, and inflammatory effects of adipose tissue [5–7]. Endogenous hormones, such as insulin-like growth factor-I (IGF-I) and free testosterone (unbound testosterone that is biologically active), have been associated with a higher risk of metabolic syndrome, cardiovascular disease, and some obesity-related cancers [8–12] and may play a role in these associations. Sex hormone-binding globulin (SHBG) is not a hormone, but it transports sex steroids in the blood by binding to these hormones, therefore regulating their bioavailability.

Previous cross-sectional studies have shown that IGF-I concentrations have an inverse U-shaped relationship with common adiposity measures, such as BMI, while SHBG, total testosterone, and free testosterone concentrations have shown inverse relationships with BMI [13, 14]. However, the use of common adiposity measures may limit previous investigations of the relationships between obesity and hormone concentrations due to their inability to measure fat in different body locations, and the limited ability to differentiate between lean and fat tissue [15]. Magnetic resonance imaging (MRI) may offer greater insight into the associations between adiposity and hormone concentrations due to its ability to differentiate fat and lean tissue in differing regions of the body (e.g., whole-body, central, site-specific) [16]. Moreover, MRI is able to measure fat stored in tissue other than the adipose tissue, such as organs and muscle, which is known as ectopic fat [17]. However, due to the high cost of using MRI to measure body composition, previous studies have been limited by small sample sizes and to the best of our knowledge, no large-scale study has assessed the associations of MRI measurements of body composition with these endogenous hormones and SHBG.

Our aim was therefore to investigate the associations of MRI measures of body composition with serum concentrations of IGF-I, testosterone, free testosterone, and SHBG in a cohort of 16,237 men. We also describe the associations of classic anthropometric measures with these hormones for comparison with the MRI measures.

## Methods

### Data sources and study setting

The UK Biobank is a prospective cohort study involving over 500,000 participants aged 40–70 years living in the UK. Between 2006 and 2010, 9.2 million residents living within a radius of 40 km of the 22 UK Biobank assessment centres across England, Wales and Scotland were contacted via National Health Service records, and 5.5% opted to participate [18]. At recruitment, participants’ blood samples were collected for biomarker estimation, and detailed baseline characteristics regarding anthropometry, demographic, social factors, and health information were recorded.

After the recruitment visit, two subsamples of the UK Biobank participants attended follow-up assessments, one for biomarker re-measurement ~5 years after the baseline assessment (*n* = ~20,000), and another for MRI imaging a median of 8 years after baseline (data available for *n* = 60,000 as of January 2022). Further details about the UK Biobank can be found elsewhere [19].

### Anthropometric measurements

#### MRI data collection (primary exposure)

In 2014, the UK Biobank began the largest multi-modal imaging study in the world, aiming to collect MRI data on 100,000 (20%) UK Biobank participants [16]. Approximately 40,000 participants (~17,000 men) had abdominal MRI imaging data available for analysis in January 2023. MRI imaging was performed following a detailed protocol using a Siemens 1.5T Tesla MAGNETOM Aera scanner (Siemens Healthineers, Erlangen, Germany) [16, 20]. Further details regarding recruitment to the imaging portion of the UK Biobank can be found in the Supplementary Methods.

Eight measures of body composition were collected via MRI and used in these analyses: visceral adipose tissue (VAT), abdominal subcutaneous adipose tissue (ASAT), total trunk adipose tissue, total adipose tissue, total abdominal adipose tissue index, liver proton density fat fraction (PDFF), total lean tissue volume (non-fat metabolically active tissue—e.g., muscle), and muscle fat infiltration (MFI). Further details regarding the definition of each MRI body composition measurement are described in the Supplementary Methods.

#### Classic anthropometric measurements

Classic anthropometric measures were collected in all UK Biobank assessments (recruitment, follow-up, and MRI imaging). These include the common adiposity measures of BMI, body fat % measured via bioimpedance (Tanita BC418MA body composition analyser), and waist circumference (WC). Waist-to-hip ratio (WHR) was calculated by dividing WC by the hip circumference measured using a tape by a trained member of staff. To compare classic anthropometric measures with those from MRI imaging data, classic anthropometric measures collected during the imaging assessment were used to evaluate their associations with the biomarkers of interest.

### Serum biomarker measurement (outcome)

At recruitment, 99.7% of participants provided a non-fasted blood sample. Samples were collected and maintained at 4 °C prior to transportation to a central laboratory in which they were processed and then stored at −80 °C for later assay measurement [21]. Serum concentrations of IGF-I were measured via chemiluminescent immunoassay (DiaSorin Liaison XL); SHBG and total testosterone concentrations were also measured using chemiluminescent immunoassays (Beckman Coulter DXI 800) [22] and serum concentrations of albumin (used to calculate free testosterone) were measured using a colourimetric assay (Beckman Coulter AU5800). An estimate of free testosterone, unbound to SHBG or albumin, was calculated using the law of mass action and participant measurements of SHBG, total testosterone, and albumin [23, 24].

Those attending the first follow-up visit between 2012–2013 provided an additional non-fasted blood sample and hormone biomarkers were re-measured [22]; these re-measurements were used in sensitivity analyses.

### Exclusion criteria

For the main analysis, participants were excluded if they reported their sex was female (*n* = 273,310) and did not have MRI imaging data (*n* = 209,700). Participants were further excluded for having no hormonal biomarker data at recruitment (*n* = 1230), no BMI data from the imaging visit (*n* = 532), prevalent malignant cancer (any cancer except non-melanoma skin cancer diagnosed before the imaging visit, *n* = 1207), taking medications at recruitment that may impact hormone levels (*n* = 161; see Supplementary Table 1), or reported sex not matching their genetic sex (*n* = 9). After applying these exclusions, a total of 16,237 men from the UK Biobank were included. A flowchart of exclusions may be found in Supplementary Fig. 1, and additional details of the exclusion criteria are included in the Supplementary Methods.

In sensitivity analyses, we used a subset of men with a second biomarker measurement from blood taken at a follow-up visit (*n* = 2681), and men with a BMI changing less than 5% between recruitment and imaging visits (*n* = 10,408).

The number of participants included in each analysis varied across the various MRI measures and biomarker combinations due to delays in assessing the MRI data for the various body compartments, resulting in missing data for some participants. As such, analyses between adiposity measures and hormone concentrations varied from 3370 to 16,237 in the number of participants included, with analyses assessing lean tissue and total adipose tissue in relation to hormone concentrations often including the lowest number of participants.

### Statistical analysis

Participant’s baseline characteristics and adiposity measures were summarised across three categories of BMI (<25.0, 25.0–29.9, ≥30.0 kg/m^2^). Hormone concentrations and liver PDFF were log-transformed to approximate a normal distribution. Pearson’s correlation coefficients (*r*) between all body composition measures and hormonal biomarkers were calculated and assessed for strong correlations (|*r*| = ≥0.80) or moderate correlations (|*r*| = ≥0.60) [25]. Histograms of the outcome and exposure variable distributions are available in Supplementary Fig. 2.

Linear regression models were used to estimate the association between measures of body composition and hormonal biomarker concentrations. Measures of body composition were categorised into quintiles to compare results with previously published studies [8, 13, 14]. Covariates were determined a priori and were included in the multivariable models if they were deemed to be a plausible confounder between adiposity and hormonal biomarker concentrations. For covariates in which responses were unknown or missing, participants were categorised into an unknown/missing category.

Minimally-adjusted linear regression models were adjusted for age at recruitment (<45, 45–49.9, 50–54.9, 55–59.9, 60–64.9, ≥65 years) and ethnicity (White, Mixed Race, Asian or Asian British, Black or Black British, other, or missing/unknown (0.3%)). Multivariable linear regression models were further adjusted for education (no educational qualifications, completion of national exam at 17, attendance of university or college, or missing/unknown (0.3%)), Townsend Deprivation Index (quintiles (most deprived to most affluent), or missing/unknown (0.1%)), region of recruitment (London, North-West England, North-Eastern England, Yorkshire & the Humber, West Midlands, East Midlands, South-East England, South-West England, Wales, or Scotland), employment status (not in paid/self-employment, in paid/self-employment), cohabitation status (not living with a partner, living with a partner), height (<170, 170–174.9, 175–179.9, 180–184.9, ≥185 cm, or missing (0.1%)), diabetes (no history of diabetes, history of diabetes, or missing/unknown (0.2%)), cigarette smoking status (never, previous smoker, current smoker, or missing/unknown (0.3%)), alcohol consumption status (non-drinker, <1, 1–9, 10–19, ≥20 g/day, or missing/unknown (0.1%)), and physical activity status (low, moderate, high, or missing/unknown (1.7%)).

Geometric mean hormonal biomarker and SHBG concentrations were estimated using predictive values from the multivariable linear regression models using the margins, fixing covariates at their means for each category. These values were then exponentiated and the ratio of the geometric mean values relative to the first quintile represent the relative geometric mean biomarker concentrations. Likelihood ratio tests (LRTs) were used to compare the *χ*^2^ from each model to a model without the exposure (body composition measure) to assess if the exposure of interest significantly added to the model. LRTs were also used to assess for non-linearity by comparing models built using categorical quintiles of the exposure to models with the quintiles modelled continuously. If LRTs were statistically significant when assessing for non-linearity then associations were deemed to be non-linear.

#### Sensitivity analyses

To account for the years between hormonal biomarker measurement at recruitment (2006–2010) and MRI imaging data (2014–2020), sensitivity analyses were performed on participants that had a repeat hormonal biomarker measurement at follow-up (2012–2013; *n* = 2681). For participants with two biomarker measurements, the mean of these two measures was taken and log-transformed to approximate a normal distribution. Moreover, to assess for long-term changes in BMI measures, and control for fluctuations in BMI which may in turn correspond to changes in MRI measures between assessments, another analysis was restricted to participants with a BMI at the imaging visit (2014–2020) within ±5% their BMI at recruitment (2006–2010; *n* = 10,408). Quintiles for each exposure were re-defined for each sensitivity analysis.

A *p* value of <0.0001 was considered statistically significant to account for the numerous comparisons. All statistical analyses were performed using STATA version 17.0.

## Results

### Participants’ characteristics

Men included in this study had a mean age of 55.3 (SD 7.6) years at recruitment, a mean BMI of 27.0 (SD 3.9) kg/m^2^ at the imaging visit and were predominately of White ethnicity (96.5%; Table 1). Men in the highest category of BMI (≥30 kg/m^2^) were more likely to have self-reported diabetes and be physically inactive. All MRI measures of adiposity (e.g., visceral adipose tissue) increased across the three BMI categories, and men with a BMI of ≥30 kg/m^2^ had lower levels of IGF-I, free testosterone, and SHBG, in comparison to those with a BMI < 25 kg/m^2^ (Table 1).

**Table 1:** Participant characteristics in up to 16,237 men from the UK Biobank stratified by total cohort and subcategories of BMI.

	Total	BMI (kg/m^2^)		
		<25.0	25.0–29.9	≥30.0
Total number of participants, *n*	16,237	5189	7870	3178
Age at recruitment (years), mean (SD)	55.3 (7.6)	55.5 (7.6)	55.4 (7.5)	54.6 (7.5)
Sociodemographic, *n* (%)				
Higher educational qualifications	12,570 (77.4)	4302 (82.9)	6002 (76.3)	2266 (71.3)
Most deprived category	2130 (13.1)	648 (12.5)	987 (12.5)	495 (15.6)
Living with partner	13,259 (81.7)	4242 (81.7)	6539 (83.1)	2478 (78.0)
Not in paid/self-employment	4666 (28.7)	1549 (29.9)	2247 (28.6)	870 (27.4)
Current cigarette smokers	1222 (7.5)	329 (6.3)	592 (7.5)	301 (9.5)
Drinking alcohol ≥20 g/day	7202 (44.4)	1974 (38.0)	3719 (47.3)	1509 (47.5)
Physically inactive	4314 (26.6)	1230 (23.7)	2053 (26.1)	1031 (32.4)
White ethnicity	15,662 (96.5)	4987 (96.1)	7583 (96.4)	3092 (97.3)
Self-reported diabetes	562 (3.5)	106 (2.0)	243 (3.1)	213 (6.7)
Region of imaging (Central England)	10,332 (63.6)	3223 (62.1)	4989 (63.4)	2120 (66.7)
Biomarker, mean (SD)				
IGF-I (nmol/L)	22.5 (5.2)	22.5 (5.0)	22.8 (5.1)	21.8 (5.5)
SHBG (nmol/L)	38.9 (15.6)	44.6 (16.5)	37.4 (14.4)	33.1 (13.9)
Total testosterone (nmol/L)	12.2 (3.5)	13.1 (3.6)	12.1 (3.4)	11.0 (3.3)
Free testosterone (pmol/L)	214.4 (56.8)	212.9 (53.9)	217.3 (57.7)	209.7 (58.5)
Anthropometric measures, mean (SD)				
Height (cm)	176.7 (6.6)	177.1 (6.7)	176.6 (6.5)	176.2 (6.6)
BMI (kg/m^2^)	27.0 (3.9)	23.1 (1.4)	27.2 (1.4)	33.0 (3.0)
Body fat (%)	25.5 (5.6)	20.7 (4.4)	26.0 (3.7)	31.8 (4.0)
Waist circumference (cm)	94.3 (10.7)	84.9 (6.3)	94.8 (6.3)	108.2 (9.1)
Waist to hip ratio	0.93 (0.06)	0.89 (0.05)	0.94 (0.05)	0.99 (0.06)
MRI measure of body composition, mean (SD)				
Visceral adipose tissue volume (L)	4.9 (2.3)	2.9 (1.4)	5.1 (1.6)	7.7 (2.0)
Abdominal subcutaneous adipose tissue volume (L)	5.8 (2.5)	3.8 (1.1)	5.8 (1.4)	9.1 (2.8)
Total abdominal adipose tissue index (L/m^2^)	3.5 (1.4)	2.2 (0.7)	3.5 (0.8)	5.4 (1.2)
Total trunk adipose tissue volume (L)	10.7 (4.4)	6.7 (2.2)	10.9 (2.4)	16.8 (3.9)
Total adipose tissue volume (L)	19.6 (6.4)	13.7 (3.3)	20.0 (3.7)	28.6 (5.3)
Total lean tissue volume (L)	28.2 (3.4)	26.4 (3.0)	28.5 (3.0)	30.7 (3.4)
Muscle fat infiltration (%)	6.7 (1.7)	5.9 (1.3)	6.8 (1.5)	7.9 (1.9)
Liver PDFF (%)	4.7 (4.8)	2.5 (2.2)	4.7 (4.4)	8.3 (6.2)

Age, sociodemographic, and biomarker data were collected at recruitment from 2006–2010. Anthropometric measures and MRI measures were collected at MRI imaging from 2014–2020.

*BMI* body mass index, *IGF-I* insulin-like growth factor-I, *n* number of participants, *PDFF* proton density fat fraction, *SD* standard deviation, *SHBG* sex hormone-binding globulin.

### Correlations between body composition measurements

Supplementary Table 2 presents the correlations between MRI measurements, classic anthropometric measurements, and hormonal biomarkers and SHBG. Most MRI adiposity measures were highly correlated with each other (*r* = 0.85–0.98), although the correlation between visceral and abdominal subcutaneous adipose tissues was smaller (*r* = 0.69). However, MRI measures of lean tissue, MFI, and liver PDFF were not strongly correlated with any other MRI measure (*r* = −0.10–0.55) or any common adiposity measure (*r* = 0.11–0.54). BMI, body fat %, and WC were highly correlated with each other (*r* = 0.77–0.86), but generally less correlated with WHR (*r* = 0.58–0.78), and highly correlated with the MRI measures of adipose tissue (*r* = 0.76–0.91).

### Associations of body composition measures with geometric mean hormonal biomarker and SHBG concentrations

**Fig. 1:**
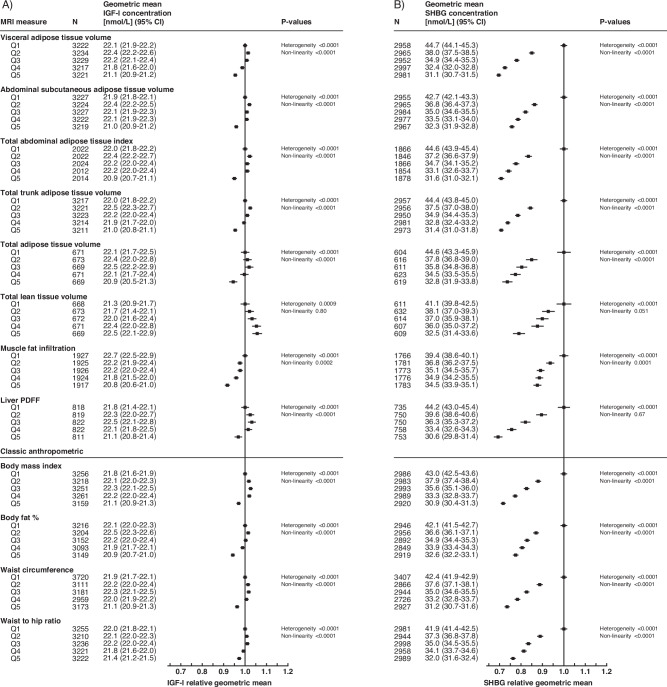
Multivariable adjusted model of body composition measures in relation to the geometric mean concentrations of IGF-I and SHBG. **A** IGF-I relative geometric mean concentrations, **B** SHBG relative geometric mean concentrations. MRI measures are found in the top portion of the figure, with classic anthropometric in the lower portion. The relative geometric mean is the ratio of log-transformed values relative to the first quintile. *P* value for heterogeneity assesses evidence of an overall association; *p* value for non-linearity assesses departure from linearity per incremental increase of exposure (significance indicates non-linearity). CI confidence interval, MRI magnetic resonance imaging, N number of participants, PDFF proton density fat fraction, Q quintile, SD standard deviation.

**Fig. 2:**
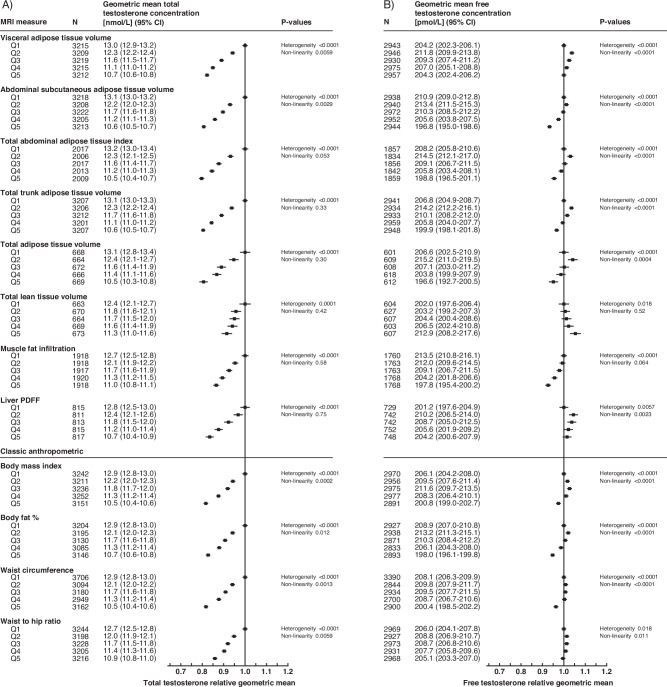
Multivariable adjusted model of body composition measures in relation to the geometric mean concentrations of total testosterone and free testosterone. **A** Total testosterone relative geometric mean concentrations, **B** free testosterone relative geometric mean concentrations. MRI measures are found in the top portion of the figure, with classic anthropometric in the lower portion. The relative geometric mean is the ratio of log-transformed values relative to the first quintile. *P* value for heterogeneity assesses evidence of an overall association; *p* value for non-linearity assesses departure from linearity per incremental increase of exposure (significance indicates non-linearity). CI confidence interval, MRI magnetic resonance imaging, N number of participants, PDFF proton density fat fraction, Q quintile, SD standard deviation.

The geometric mean concentrations of IGF-I, SHBG, total testosterone, and free testosterone by quintiles of body composition measures from fully adjusted linear regression models are presented in Figs. 1 and 2 (see minimally-adjusted models in Supplementary Figs. 3 and 4). The addition of covariates in multivariable models generally did not change the associations between body composition measures and IGF-I, SHBG, total testosterone, and free testosterone (Figs. 1 and 2 and Supplementary Figs. 3 and 4).

Most MRI adiposity measurements, and all common adiposity measurements, showed a significant inverse U- or J-shaped association with the geometric mean concentrations of IGF-I and free testosterone (Figs. 1A and 2B). Lean tissue had a positive linear association with free testosterone and IGF-I concentrations, with a 1.2 nmol/L (~5%) higher geometric mean IGF-I concentration in the highest quintile in comparison to the lowest quintile (Fig. 1A). MFI had an inverse linear association with IGF-I and free testosterone concentrations, respectively (Figs. 1A and 2B).

All measures of body composition showed a significant inverse relationship with the geometric mean concentration of SHBG and total testosterone (Figs. 1B and 2A). For most body composition associations with SHBG, the relative geometric mean changed more between the lowest and second-lowest quintiles (e.g., 10–15% lower concentrations for MRI measures of adiposity) than between subsequent quintiles (Fig. 1B). Liver PDFF and SHBG showed the strongest overall association, with a 31% lower geometric mean SHBG in the highest quintile in comparison to the lowest quintile (Fig. 1B).

### Sensitivity analysis

Sensitivity analyses restricted to 2681 men with repeat biomarker measures and 10,408 men with stable BMI can be found in Supplementary Figs. 5–8, respectively. Free testosterone broadly showed weaker associations with measures of body composition in men with repeat biomarker measurements (Supplementary Fig. 6B). The remaining associations were very similar to those observed in the main analyses, although larger confidence intervals were observed due to the smaller number of men included.

## Discussion

This observational analysis used gold-standard MRI measurements of body composition to assess the relationship with serum hormonal biomarker and SHBG concentrations and showed that MRI and classic anthropometric measures of adiposity each had similar associations with serum concentrations of IGF-I, SHBG, total testosterone and free testosterone, although associations with ectopic fat were weaker. Specifically, MRI measures of adiposity had inverse U- or J-shaped associations with IGF-I and free testosterone concentrations—with the exception of MFI, which was linearly inversely associated with IGF-I and free testosterone concentrations—and inverse associations with SHBG and total testosterone concentrations. Lean tissue was positively associated with IGF-I and free testosterone concentrations. To our knowledge, this is the largest study to date assessing the associations of MRI measurements of body composition with hormonal biomarkers and SHBG in men.

Our results showed that common adiposity measures (i.e., BMI, body fat % and WC) were highly correlated with MRI measures of central and total adiposity (e.g., VAT, ASAT) which has been shown in other observational studies of ~40–200 male participants from the UK [26–28]. MRI measures of lean tissue volume and ectopic fat (i.e., MFI and liver PDFF) had weaker correlations with the common adiposity measures. The latter may be particularly important as common measures of adiposity do not adequately capture the proportion of ectopic fat in the body, which may have independent associations with disease outcomes.

In our study, common and MRI measurements of adiposity were associated with concentrations of IGF-I in an inverse U- or J-shaped manner, which has been previously reported for BMI and WC in cross-sectional studies of 400–16,000 men [13, 29, 30]. To the best of our knowledge limited research has explored MRI adiposity measures with IGF-I concentrations in adult men. One study of 14 men with acromegaly, a condition where individuals have markedly high IGF-I concentrations, suggested lower ASAT and VAT measured by MRI among these men in comparison to controls [31]. However, in contrast to our study, a small observational study of 267 men found no association between IGF-I and adiposity (VAT, ASAT, total fat mass) as assessed through computed tomography [32]. Two further small studies of 17 and 33 men observed no significant changes in IGF-I concentrations following bariatric surgery and subsequent weight loss [33]. IGF-I stimulates cell differentiation and proliferation in numerous tissues, and existing research has suggested that growth hormone therapy, which stimulates IGF-I production, reduces VAT [34]; however, it is not clear the direction of effect and the magnitude is not large. Further research into other growth factors, IGF-II, and IGF-binding proteins—which may be influenced by body composition—is needed to clarify these associations as well as their potential mechanisms of action [13, 29, 30, 35].

Both MRI and common measures of adiposity were inversely associated with free testosterone concentrations in a J-shaped manner. We also observed differences in the association patterns between total testosterone and free testosterone with body composition measurements. Having greater adipose tissue will lead to higher aromatase activity [36], which may lead to androgens being converted to oestrogens, thus resulting in less circulating testosterone. As well, as observed in this study and others, SHBG is greatly reduced with greater adiposity [14, 37], which will influence free testosterone concentrations.

Similar to this study, a cross-sectional study of 406 healthy young men found MRI measures of VAT and ASAT were inversely associated with total testosterone and free testosterone [37]. In a randomised controlled trial of 60 men, transdermal testosterone patches were found to decrease VAT and ASAT measured by MRI [38], and in a study of 45 men with type 2 diabetes, bariatric surgery caused a decrease in MRI-measured VAT that was inversely associated with total testosterone levels [39]. Moreover, in a study of 1453 men from Korea, ASAT, but not VAT, measured by computed tomography was inversely associated with testosterone [40]. Both free and circulating testosterone has been previously inversely associated with common adiposity measures in numerous studies of upwards of 12,000 men [14, 41]. Testosterone is responsible for secondary sex-related characteristics—stimulating muscle growth, erythropoiesis, and cell growth in the prostate—and has been implicated in prostate cancer risk [42]. It remains unclear why weaker associations were observed in sensitivity analyses for men with repeat biomarker measurements. This may be due to the smaller sample size in the sensitivity analysis, an effect of temporality, or overall weaker associations, as free testosterone had the fewest significant associations of the assessed hormonal biomarkers.

We also observed both MRI and classic anthropometric measures were inversely associated with SHBG, which is in line with previous studies [14, 37]. Specifically, in one cross-sectional study of 406 Danish men, MRI measured VAT and ASAT were inversely associated with SHBG concentrations [37]. We also observed that liver PDFF was linearly inversely associated with SHBG and was the strongest association observed in this study. SHBG is produced by the liver and some evidence suggests that high liver fat results in a reduction in circulating SHBG [43]. Furthermore, low SHBG levels have been observed in patients with non-alcoholic fatty liver disease [44], and it is possible that higher ectopic fat in the liver inhibits liver function and reduces SHBG production.

Our findings also showed that MFI had an inverse association with all hormonal biomarkers and SHBG concentrations. To our knowledge, limited research has explored MFI’s association with hormones, with one cross-sectional analysis of 1453 Korean men finding no association between testosterone concentrations and intermuscular fat [40]. Further work should potentially explore these associations.

In this study, the associations of lean tissue with hormonal biomarkers were different to those observed for adiposity measurements, as it showed a positive linear association between lean tissue volume and serum concentrations of IGF-I and free testosterone. This aligns with observational findings showing a positive association between IGF-I and lean tissue in 776 Danish men aged 20–29 years [45], and free testosterone concentrations and muscle mass measured with dual-energy x-ray absorptiometry (DXA) [46, 47]. Testosterone and IGF-I are known to increase muscle mass [34, 47–51], and the positive linear relationships we observed are likely a result of these hormones supporting/stimulating lean tissue growth.

Strengths of our analyses include the large cohort size and detailed phenotyping data available. The UK Biobank is the largest prospective cohort study to contain biomarker measurements for practically the entire cohort, and secondary biomarker and MRI measurements on a sub-cohort. Moreover, the UK Biobank has collected detailed sociodemographic and lifestyle factors on all participants, thus we were able to adjust analyses for potential confounders.

Limitations of this analysis include the unclear directions of causal relationships underlying the associations observed between body-composition and hormonal biomarkers, as body composition and hormones are related in a reciprocal manner. Although BMI was used as a common index of adiposity because it is strongly correlated with both body fat percentage and adiposity in general population studies, it does not differentiate between fat and lean mass. The reliance upon biomarker measurements taken several years earlier than the MRI imaging is also a limitation as the median time between recruitment and imaging was eight years (range 3.8–12.6 years). However, our sensitivity analyses restricted to participants with repeat biomarker measurements (median of 3.5 years between measurement and imaging, range of 1.0–6.9 years) and participants with stable BMI showed that the results were largely unchanged. The UK Biobank used chemiluminescent immunoassays to measure circulating androgens, which has analytical limitations in comparison to gold standard methods [52], however, for large epidemiological purposes, this method accurately ranks participants and therefore can be used to compare between participants. Moreover, the calculation of free testosterone using the law of mass action may deviate from the true concentration, however, it has been found to correlate strongly with direct laboratory measures [53]. Additionally, results may not be generalisable, including to other racial and ethnic groups; the UK Biobank is not fully representative of the contemporary UK population as participants are predominately White, and are also comparatively healthier and from a higher socioeconomic status [18], with imaging done in a subsample who are likely to be more health-conscious. However, the direction of relationships is likely to remain the same [54]. While ~30,000 men in UK Biobank have undergone MRI imaging, the processing delay in quantifying their body composition means that only around half (17,614 men) were available for analysis (before further exclusion criteria). Due to numerous comparisons, chance findings are still possible, however, we used a conservative *p* value of 0.0001 to signify statistical significance which reduces the chance of a type I error. As well, using body fat percentage may be a potentially flawed measure [55] and these results should be interpreted cautiously. Only details of covariates collected at recruitment were used for model adjustment, aiming to adjust for any association they may have had on the initial biomarker measurements. There is also potential for residual confounding by imperfect measurement of confounders or not adjusting for unmeasured covariates, therefore causality cannot be determined. Future research exploring the interplay between genetic factors, body composition, and hormonal biomarkers may provide additional insights into the complex association between obesity and health outcomes. Finally, additional research is needed to examine these associations in women. However, such analyses would require careful consideration of factors such as changes in menopausal status between blood collection and the time of imaging data collection, which are likely to affect hormonal concentrations during this period.

## Conclusion

Our results indicate that both common (e.g., BMI) and MRI measures of total and central adiposity (e.g. VAT, ASAT) were highly correlated and showed similar inverse associations with concentrations of hormonal biomarkers and SHBG, with associations for ectopic fat being weaker. We also found novel associations of MFI and lean tissue volume with some of these hormonal biomarkers and further research should explore this in relation to the risk of cancer and other health conditions.

## Supplementary information

Supplementary Materials

## Data Availability

The UK Biobank is an open-access resource; further information on accessing these data may be found at https://www.ukbiobank.ac.uk.
