# Supplementary material for: Associations of body composition measures with circulating insulin-like growth factor-I, testosterone, and sex hormone-binding globulin concentrations in 16,000 men
Source: Int J Obes (Lond). 2024 Oct 21;48(12):1809–17. doi: 10.1038/s41366-024-01633-0 (PMC11584381; doi:10.1038/s41366-024-01633-0)
Supplement: Supplementary file 1 — Supplementary Materials [file 41366_2024_1633_MOESM1_ESM.docx]

**Supplementary Methods and Materials**

**Associations of** **body composition measures with circulating insulin-like growth factor-I, testosterone, and sex hormone-binding globulin concentrations in 16,000 men**

Matthew C. Hynes^1*^, Cody Z. Watling^1*^, Yashvee Dunneram^1^, Tim J. Key^1^, Aurora Perez-Cornago^1^

^1^ Cancer Epidemiology Unit, Nuffield Department of Population Health, University of Oxford, Oxford, UK

[Supplementary Methods 4](#_Toc175759875)

[Additional covariate information 4](#_Toc175759876)

[Age at recruitment 4](#_Toc175759877)

[Alcohol consumption status 4](#_Toc175759878)

[Cigarette smoking status 4](#_Toc175759879)

[Cohabitation status 5](#_Toc175759880)

[Diabetes 5](#_Toc175759881)

[Education 5](#_Toc175759882)

[Employment status 5](#_Toc175759883)

[Ethnicity 6](#_Toc175759884)

[Height 6](#_Toc175759885)

[Physical activity status 6](#_Toc175759886)

[Region of recruitment 7](#_Toc175759887)

[Townsend Deprivation Index 7](#_Toc175759888)

[Additional data information 8](#_Toc175759889)

[Imaging study 8](#_Toc175759890)

[MRI body composition measures 8](#_Toc175759891)

[References 9](#_Toc175759892)

[Exclusion criteria 10](#_Toc175759893)

[Female sex 10](#_Toc175759894)

[MRI imaging data 10](#_Toc175759895)

[Hormonal biomarker data 10](#_Toc175759896)

[Body mass index data 10](#_Toc175759897)

[Prevalent malignant cancer diagnosis 10](#_Toc175759898)

[Medications 11](#_Toc175759899)

[Genetic sex mismatch 11](#_Toc175759900)

[Supplementary Tables 12](#_Toc175759901)

[Supplementary Table 1. Alphabetical list of excluded hormonal medications reported as their UK Biobank entry. 12](#_Toc175759902)

[Supplementary Table 2. Pearson’s correlation between common anthropometric measures, MRI measures, and log-transformed hormonal biomarker concentrations. 13](#_Toc175759903)

[Supplementary Figures 14](#_Toc175759904)

[Supplementary Figure 1. Participant exclusion criteria for final sample selection. 14](#_Toc175759905)

[Supplementary Figure 2. Histogram of outcome variables (A-D), and exposure variables (MRI measures of body composition (E-L), classic anthropometric measures (M-P)). 15](#_Toc175759906)

[Supplementary Figure 3. Minimally adjusted models of body composition measures in relation to (A) IGF-I and (B) SHBG geometric mean concentrations (n=16,237). 16](#_Toc175759907)

[Supplementary Figure 4. Minimally adjusted models of body composition measures in relation to (A) total testosterone and (B) free testosterone geometric mean concentrations (n=16,237). 17](#_Toc175759908)

[Supplementary Figure 5. Multivariable adjusted models comparing body composition measures to the mean of the baseline and repeated biomarker measurements for (A) IGF-I and (B) SHBG concentrations (n=2,681). 18](#_Toc175759909)

[Supplementary Figure 6. Multivariable adjusted models comparing body composition measures using the mean of the baseline and repeated biomarker measurements for (A) total testosterone and (B) free testosterone (n=2,681). 19](#_Toc175759910)

[Supplementary Figure 7. Multivariable adjusted models comparing body composition measures to (A) IGF-I and (B) SHBG concentrations restricted to participants where BMI changed less than 5% between recruitment and imaging visits (n=10,408). 20](#_Toc175759911)

[Supplementary Figure 8. Multivariable adjusted models comparing body composition measures to (A) total testosterone and (B) free testosterone concentrations restricted to participants where BMI changed less than 5% between recruitment and imaging visits (n=10,408). 21](#_Toc175759912)

# Supplementary Methods

## Additional covariate information

### Age at recruitment

Participant’s age at recruitment was calculated using the participant’s date of birth and date of recruitment into the UKB. Participant were grouped by age into one of six categories: <45, 45-49.9, 50-54.9, 55-59.9, 60-64.9, and ≥65 years.

### Alcohol consumption status

Participant alcohol consumption in grams per day was assessed through the conversion of participant-reported weekly and monthly alcoholic drink consumption. Participants were asked about their average weekly and monthly consumption of ‘pints of beer or cider,’ ‘glasses of red wine,’ ‘glasses of white wine or champagne,’ ‘glasses of fortified wine,’ ‘measures of spirits or liqueurs,’ and ‘glasses of other alcoholic drinks’ in touchscreen questionnaires. The grams of alcohol in each drink was estimated at twenty grams for one pint of cider or beer and ten grams for one glass of any other alcoholic beverage. The average daily grams of alcohol consumed for each type of alcoholic drink was calculated by dividing the average grams per week by seven, and by dividing the average grams per month by 30.4375. A participant’s alcohol consumption in grams per day was estimated by summing the average daily consumption for each type of alcoholic drink. Average daily consumption calculated from average weekly consumption was primarily used to estimate the average daily grams of alcohol consumed, and in cases where this value was missing, the average daily consumption calculated from monthly consumption was used instead. Participant’s alcohol consumption was categorised as <1 g/day, 1-10 g/day, 10-20 g/day, >20 g/day, non-drinker, or missing/unknown/prefer not to answer.

### Cigarette smoking status

Participants were asked two questions to assess their cigarette smoking status, ‘do you smoke tobacco now?’ and, ‘in the past, how often have you smoked tobacco?’ Participant cigarette smoking status was categorised as never, previous, or current smoker. Participants that self-reported smoking at least one cigarette per day when asked, ‘about how many cigarettes do you smoke on average each day?’ were also categorised as current smokers. Participants were categorised as ‘missing/unknown/prefer not to answer’ if they had missing data, reported their smoking status as ‘prefer not to answer,’ or the average number of cigarettes smoked each day as ‘unknown.’

### Cohabitation status

Cohabitation status may approximate marital status and participants were categorised as ‘not living with a partner’ or ‘living with a partner’. Participants were asked ‘how are the other people who live with you related to you?’ and those that selected ‘husband, wife or partner’ were categorised as living with a partner. Participants categorised as not living with a partner were not necessarily living alone, and also included participants that identified as living with a ‘son and/or daughter (include step-children),’ ‘brother and/or sister,’ ‘mother and/or father,’ ‘grandparent,’ ‘grandchild,’ ‘other related,’ or ‘other unrelated.’

### Diabetes

To determine a participant’s history of diabetes, participants were asked, ‘has a doctor ever told you that you have diabetes?’ and presented with the options ‘yes,’ ‘no,’ ‘do not know,’ and ‘prefer not to answer.’ Participants had their self-reported diabetes status categorised as a history of diabetes, no history of diabetes, and missing/unknown/prefer not to answer. No distinction was made between type I and type II diabetes.

### Education

A participant’s education was categorised as the highest educational qualification completed. Participants were asked, ‘which of the following qualifications do you have?’ and presented with the options ‘College or University degree,’ ‘A levels/AS levels or equivalent,’ ‘O levels/GCSEs or equivalent,’ ‘CSEs or equivalent,’ ‘NVQ or HND or HNC or equivalent,’ ‘other professional qualifications eg: nursing, teaching,’ ‘none of the above,’ and ‘prefer not to answer.’ Participant’s education was categorised as higher education, A-levels, no educational qualifications/GSE/CSE, and missing/prefer not to answer. *Higher education* is considered education beyond secondary education and includes professional qualifications (National Vocational Qualification (NVQ), Higher National Diploma (HND), or Higher National Certificate (HNC)) and university or college degrees. *A-levels* (advanced levels) includes participants that completed their A-levels. *No educations qualifications/GSE/CSE* includes participants with no educational qualifications and those with an O-level result (ordinary level), Certificate of Secondary Education (CSE), or General Certificate of Secondary Education (GCSE).

### Employment status

The employment status of each participant was categories as ‘not in paid/self-employment’ or ‘in paid/self-employment.’ Participants were asked ‘which of the following describes your current situation?’ and participants that selected ‘in paid employment or self-employed’ were categorised as such. Participants that selected ‘retired,’ ‘looking after home and/or family,’ ‘unable to work because of sickness or disability,’ ‘unemployed,’ ‘doing unpaid or voluntary work,’ ‘full or part-time student,’ or ‘none of the above’ were not considered to be in paid or self-employment.

### Ethnicity

Ethnicity was reported in the touchscreen questionnaire and divided into 5 categories: White, Mixed Race, Asian or Asian British, Black or Black British, other, or missing/unknown. *White* included participants that indicated their ethnic background as ‘White,’ ‘British,’ ‘Irish,’ or ‘Any other white background.’ *Mixed Race* was composed of participants indicating their ethnic background as ‘Mixed,’ ‘White and Black Caribbean,’ ‘White and Black African,’ ‘White and Asian,’ or ‘Any other mixed background.’ *Asian or Asian British* includes participants that identified as ‘Asian or Asian British,’ ‘Indian,’ ‘Pakistani,’ ‘Bangladeshi,’ ‘Chinese,’ or ‘Any other Asian background.’ *Black or Black British* included participants identifying as ‘Black or Black British,’ ‘Caribbean,’ ‘African,’ or ‘Any other Black background.’ *Other* included participants that identified their ethnic background as an ‘Other ethnic group.’

### Height

Participant height was estimated while standing using a Seca 202 device. Height was recorded in cm and participants were categorised as <170, 170-174.9, 175-179.9, 180-184.9, ≥185 cm, or missing.

### Physical activity status

Participant physical activity status was categorised as low, moderate, and high, corresponding to 0-9.9, 10-49.9, and 50-350 metabolic equivalent task (MET) per week, based on IPAQ (International Physical Activity Questionnaire) standards. The number of days of reported vigorous physical activity, moderate physical activity, and walking was multiplied by the reported duration of each activity. Participants with more than 21 hours of each physical activity per week were truncated at 21 hours (3 hours per day). These values were then multiplied by 7.0, 3.0, and 2.3 for the respective MET conversions for vigorous activity, moderate activity, and walking. Weekly METs were summed, and participants were categorised to their physical activity status.

### Region of recruitment

Participant region of recruitment was defined as London, Wales, North-West England, North-East England, Yorkshire & the Humber, West Midlands, East Midlands, South-East England, South-West England, or Scotland. Several individual assessment centres may be found within each region, and regions are composed of the following centres.

London: Barts Assessment Centre, Hounslow Assessment Centre, Barking Assessment Centre, Croydon Assessment Centre

Wales: Wrexham Mobile Assessment Centre, Swansea Mobile Assessment Centre, Cardiff Assessment Centre

North-West England: Altrincham Assessment Centre, Manchester Assessment Centre, Liverpool Assessment Centre, Manchester North Assessment Centre

North-East England: Newcastle Assessment Centre, Middlesbrough Assessment Centre

Yorkshire & Humber: Leeds Assessment Centre, Sheffield Assessment Centre

West Midlands: Stoke Assessment Centre, Birmingham Assessment Centre

East Midlands: Nottingham Assessment Centre, Leicester Assessment Centre

South-East England: Oxford Assessment Centre, Reading Assessment Centre

South-West England: Bristol Assessment Centre

Scotland: Glasgow Assessment Centre, Edinburgh Assessment Centre

### Townsend Deprivation Index

Each participants postcode was used to assign a score derived from the national census output areas. This score represents the Townsend Deprivation Index at recruitment into the UKB and was categorised into quintiles.

## Additional data information

### Imaging study

Invitation to participate in the imaging study had a 31% response rate and was extended via email and post to UKB participants living near one of four imaging centers in England. MRI imaging was performed at four sites in England: Stockport (Central), Newcastle-upon-Tyne (North), Reading (South-East), and Bristol (South-West). Participants were first recruited from the Central region in 2014, followed by the Northern, South-East, and South-West regions in 2017, 2018, and 2020, respectively [1]. The Central region currently represents the majority of available imaging data. As of November 2022, more than 60,000 participants have been imaged and participants are still being recruited.

### MRI body composition measures

Imaging covered the area from the clavicles to the distal femoral epicondyles (neck-to-knee) and took approximately 6 minutes to complete [2]. The assessment and quantification of body composition from the MRI imaging data was performed in AMRA Profiler^TM^ (AMRA AB, Linköping, Sweden) [3]. Each body composition measure had different anatomical definitions for the purposes of MRI imaging and data quantification [2, 4].

- *Visceral adipose tissue* (VAT) is adipose tissue found within the abdomen, which is the space enclosed by the abdominal skeletal muscles and vertebral column/posterior back muscles.
- *Abdominal subcutaneous adipose tissue* (ASAT) is the adipose tissue below the skin and superior to the inner abdominal cavity within the abdominal region, ranging from the top of the femoral head to the top of thoracic vertebrae T9.
- *Total trunk adipose tissue* is the total volume of fat in the abdomen, estimated through the addition of VAT and ASAT. Total adipose tissue includes additional fat below the abdominal region and is measured from the top of the T9 vertebrae to the bottom of the thigh muscle.
- *Total abdominal adipose tissue index* accounts for height and is the total abdominal fat (VAT + ASAT) divided by the square of the participant’s height.
- *Liver proton density fat fraction (PDFF)* is an estimate of fat content in the liver, calculated as the average of 3-9 regions of the liver.
- *Total lean tissue* is the volume of lean tissue (non-fat tissue excluding bone) from the top of vertebrae T9 to the bottom of the thigh muscle.
- *Muscle fat infiltration* (MFI) is the fraction of fat within the viable anterior thigh muscle tissue (quadriceps femoris, sartorius, and tensor fascia latae).

*References*

1. Littlejohns TJ, Holliday J, Gibson LM, Garratt S, Oesingmann N, Alfaro-Almagro F, et al. The UK Biobank imaging enhancement of 100,000 participants: rationale, data collection, management and future directions. Nat Commun. 2020;11:2624.

2. West J, Dahlqvist Leinhard O, Romu T, Collins R, Garratt S, Bell JD, et al. Feasibility of MR-Based Body Composition Analysis in Large Scale Population Studies. PLOS ONE. 2016;11:e0163332.

3. Borga M, Thomas EL, Romu T, Rosander J, Fitzpatrick J, Dahlqvist Leinhard O, et al. Validation of a fast method for quantification of intra-abdominal and subcutaneous adipose tissue for large-scale human studies: Quantification of IAAT and ASAT. NMR Biomed. 2015;28:1747–53.

4. Leinhard OD, Johansson A, Rydell J, Smedby O, Nystrom F, Lundberg P, et al. Quantitative abdominal fat estimation using MRI. In: 2008 19th International Conference on Pattern Recognition. Tampa, FL, USA: IEEE; 2008. p. 1–4.

## Exclusion criteria

### Female sex

On initial recruitment into the UKB, participant sex was collected from medical records and participants were able to update this field. Participants identified as female from the combination of medical records and self-report were excluded from the analyses. This is due to the body composition and hormonal biomarker associations of interest pertaining most importantly to the prostate.

### MRI imaging data

Participants were required to have data for a minimum of one out of the eight MRI measures of interest and were only excluded if data was missing for all eight measures; therefore, some participants may have data missing for 1-7 of the MRI measures.

### Hormonal biomarker data

Data for biomarker measures may be missing for several reasons, such as data not returned, values below the reportable limit, or problems in processing. Participants with extreme or unreliable biomarker data, which was defined as logarithmically transformed values lying outside upper and lower fences, had their data designated as ‘missing’. The upper and lower fences are defined as Q3+(3*IQR) and Q1-(3*IQR), respectively. Participants were required to have data for a minimum of one biomarker (IGF-I, SHBG, total testosterone, or free testosterone) from recruitment, and were excluded if data was missing for all four biomarkers; therefore, participants may have data missing for 1-3 hormonal biomarkers at recruitment.

### Body mass index data

Measures of body mass index (BMI) were taken at each UKB visit; any missing BMI measures were replaced with BMI measures calculated from the participant weight and height at the same assessment visit. BMI measurements from the MRI imaging assessment were used for the main analyses, and participants without a BMI measurement from this instance were excluded.

### Prevalent malignant cancer diagnosis

Malignant cancers were defined by ICD-9 codes 140-208 and ICD-10 codes beginning with ‘C’. ‘Prevalent’ was considered a cancer diagnosis before the date of their MRI imaging assessment, and participants with a prevalent malignant cancer diagnosis were excluded from the analysis. Participants with prevalent non-melanoma skin cancer (ICD-9 174, ICD-10 C44) were not excluded.

### Medications

Participants underwent a verbal interview during the baseline UKB visit and medication usage was documented. Participants identified as taking medications at baseline that may impact levels of hormonal biomarkers were excluded from analysis.

### Genetic sex mismatch

At recruitment, participants had their genetic sex determined through genotyping. Participants with a confirmed genetic sex that differs (mismatch) from their sex as indicated at recruitment were excluded.

# Supplementary Tables

| Supplementary Table 1. Alphabetical list of excluded hormonal medications reported as their UK Biobank entry. | |
| --- | --- |
| Medication | Medication |
| Androcur 50mg tablet | Norditropin(epr) 12iu(4mg) injection |
| Andropatch 2.5mg/24hours transdermal patch | Octreotide |
| Androstanazol | Pegvisomant |
| Avodart 500µg capsule | Primoteston Depot 250mg/1ml oily injection |
| Bicalutamide | Proviron 25mg tablet |
| Buserelin | Propecia 1mg tablet |
| Buserelin product | Proscar 5mg tablet |
| Casodex 50mg tablet | Prostap SR 3.75mg injection |
| Conjugated oestrogens 0.3mg / Medroxyprogesterone 1.5mg tab | Provera 2.5mg tablet  Restandol 40mg capsule |
| Cyproterone | Saizen(rmc) 4iu(1.33mg) injection |
| Cyproterone acetate + ethinylestradiol | Sandostatin 50µg /1ml injection |
| Cyproterone acetate + ethinyloestradiol | Somatonorm 4iu injection |
| Danazol | Somatrem |
| Deca-Durabolin 25mg/1ml oily injection | Somatropin |
| Decapeptyl SR 3mg injection (pdr for recon)+diluent | Somatropin 12iu injection  Somatuline LA 30mg injection |
| Depo-Provera 50mg/1ml injection | Somavert 10mg injection |
| Depostat 200mg/2ml oily injection | Stanozolol |
| Dutasteride | Stromba 5mg tablet |
| Finasteride | Suprefact 100µg nasal spray |
| Flutamide | Sustanon 100 oily injection |
| Genotropin 12iu multidose injection | Testoderm 6mg/24hours transdermal patch |
| Gestanin 5mg tablet | Testogel 50mg gel 5g sachet |
| Gonapeptyl Depot 3.75mg injection | Testosterone product |
| Goserelin | Testotop TTS 15mg transdermal patch |
| Goserelin product | Triptorelin |
| Humatrope(rbe) 4iu(1.3mg) injection | Virormone 10mg/1ml injection |
| Lanreotide | Virormone-oral 5mg tablet |
| Leuprorelin | Yohimbine/Pemoline/Methyltestosterone |
| Medroxyprogesterone | Zoladex 3.6mg implant |
| Mesterolone |  |
| Methyltestosterone product |  |
| Nebido 1000mg/4ml solution for injection |  |

| Supplementary Table 2. Pearson’s correlation between classic anthropometric measures, MRI measures, and log-transformed hormonal biomarker concentrations. | | | | | | | | | | | | | | | | |
| --- | --- | --- | --- | --- | --- | --- | --- | --- | --- | --- | --- | --- | --- | --- | --- | --- |
|  | BMI | Body fat % | WC | WHR | VAT | ASAT | Total abd. AT | Total trunk AT | Total AT | Total lean tissue | MFI | Liver PDFF | IGF-I | SHBG | Test. | Free Test. |
| *Classic anthropometric measures* | | |  |  |  |  |  |  |  |  |  |  |  |  |  |  |
| BMI | 1.00 |  |  |  |  |  |  |  |  |  |  |  |  |  |  |  |
| Body fat % | 0.77 | 1.00 |  |  |  |  |  |  |  |  |  |  |  |  |  |  |
| WC | **0.86** | 0.77 | 1.00 |  |  |  |  |  |  |  |  |  |  |  |  |  |
| WHR | 0.58 | 0.59 | 0.78 | 1.00 |  |  |  |  |  |  |  |  |  |  |  |  |
| *MRI measures* |  |  |  |  |  |  |  |  |  |  |  |  |  |  |  |  |
| VAT | 0.78 | 0.76 | **0.80** | 0.67 | 1.00 |  |  |  |  |  |  |  |  |  |  |  |
| ASAT | **0.85** | 0.79 | **0.82** | 0.53 | 0.69 | 1.00 |  |  |  |  |  |  |  |  |  |  |
| Total abd. AT | **0.91** | **0.87** | **0.87** | 0.67 | **0.90** | **0.90** | 1.00 |  |  |  |  |  |  |  |  |  |
| Total trunk AT | **0.89** | **0.84** | **0.88** | 0.65 | **0.91** | **0.93** | **0.98** | 1.00 |  |  |  |  |  |  |  |  |
| Total AT | **0.87** | **0.84** | **0.89** | 0.61 | **0.85** | **0.94** | **0.95** | **0.97** | 1.00 |  |  |  |  |  |  |  |
| Total lean tissue | 0.47 | 0.11 | 0.42 | 0.12 | 0.24 | 0.32 | 0.20 | 0.31 | 0.34 | 1.00 |  |  |  |  |  |  |
| MFI | 0.45 | 0.54 | 0.48 | 0.40 | 0.51 | 0.43 | 0.53 | 0.51 | 0.55 | -0.10 | 1.00 |  |  |  |  |  |
| Liver PDFF | 0.46 | 0.43 | 0.44 | 0.38 | 0.54 | 0.39 | 0.51 | 0.51 | 0.45 | 0.18 | 0.21 | 1.00 |  |  |  |  |
| *Hormonal biomarkers* | |  |  |  |  |  |  |  |  |  |  |  |  |  |  |  |
| IGF-I | -0.07 | -0.14 | -0.08 | -0.09 | -0.11 | -0.07 | -0.11 | -0.10 | -0.10 | 0.14 | -0.21 | -0.10 | 1.00 |  |  |  |
| SHBG | -0.29 | -0.20 | -0.25 | -0.21 | -0.29 | -0.23 | -0.28 | -0.28 | -0.25 | -0.20 | -0.01 | -0.27 | -0.21 | 1.00 |  |  |
| Testosterone | -0.25 | -0.24 | -0.24 | -0.19 | -0.25 | -0.24 | -0.28 | -0.27 | -0.28 | -0.05 | -0.17 | -0.19 | -0.03 | 0.59 | 1.00 |  |
| Free Testosterone | -0.04 | -0.10 | -0.06 | -0.04 | -0.04 | -0.09 | -0.09 | -0.07 | -0.11 | 0.12 | -0.19 | -0.01 | 0.15 | -0.14 | 0.70 | 1.00 |
| Correlations >0.80 are indicated in bold.  Abbreviations: abd., abdominal; ASAT, abdominal subcutaneous adipose tissue; AT, adipose tissue; BMI, body mass index; IGF-I, insulin-like growth factor I; MFI, muscle fat infiltration; PDFF, proton density fat fraction; SHBG, sex hormone-binding globulin; VAT, visceral adipose tissue; WC, waist circumference; WHR, waist to hip ratio | | | | | | | | | | | | | | | | |

# Supplementary Figures

**UK Biobank**

502,386 participants

**Sex exclusions:**

273,310 female participants

**229,076 males**

**Measurement exclusions:**

- 209,700 no MRI data
- 1,230 no biomarker data
- 532 no BMI

**17,614 males**

**Health exclusions:**

- 1,207 prevalent cancer
- 161 on hormone altering medication
- 9 genetic sex was female

**Final sample**

**16,237**

## Supplementary Figure 1. Participant exclusion criteria for final sample selection.

Supplementary Figure 2. Histogram of outcome variables (A-D), and exposure variables (MRI measures of body composition (E-L), classic anthropometric measures (M-P)).


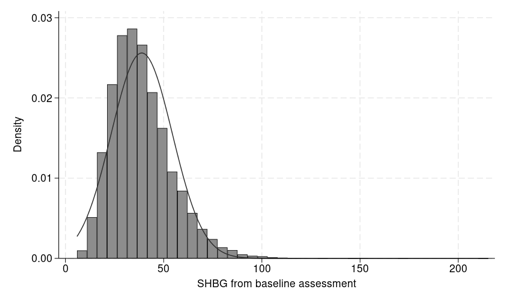

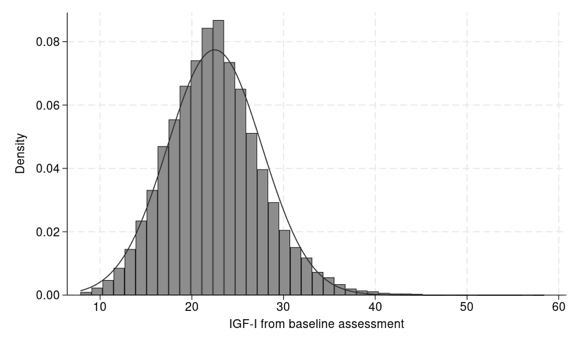

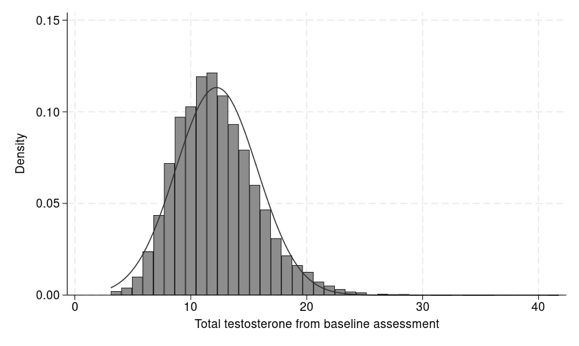

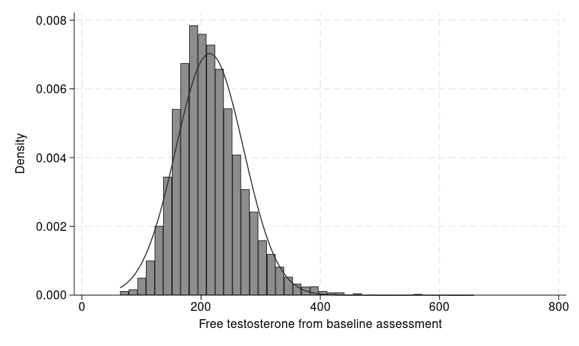

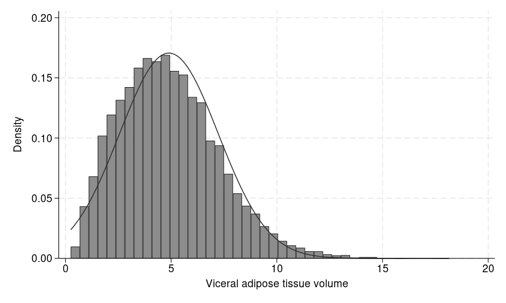

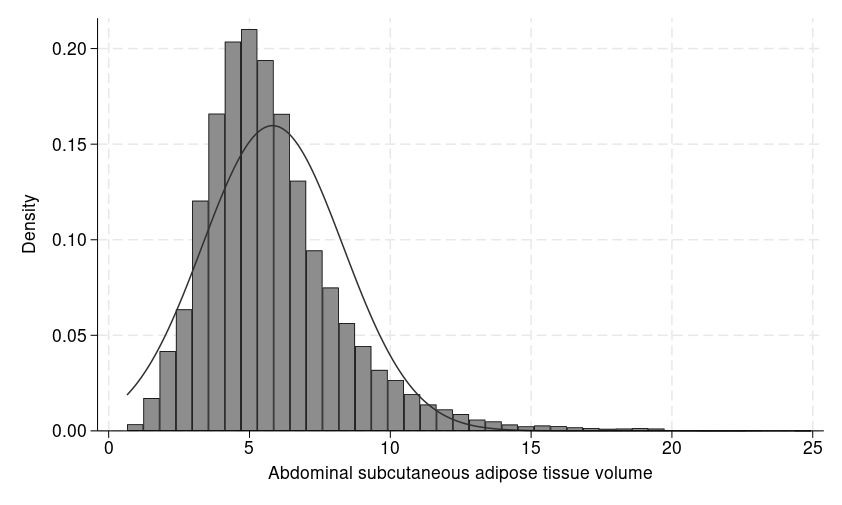

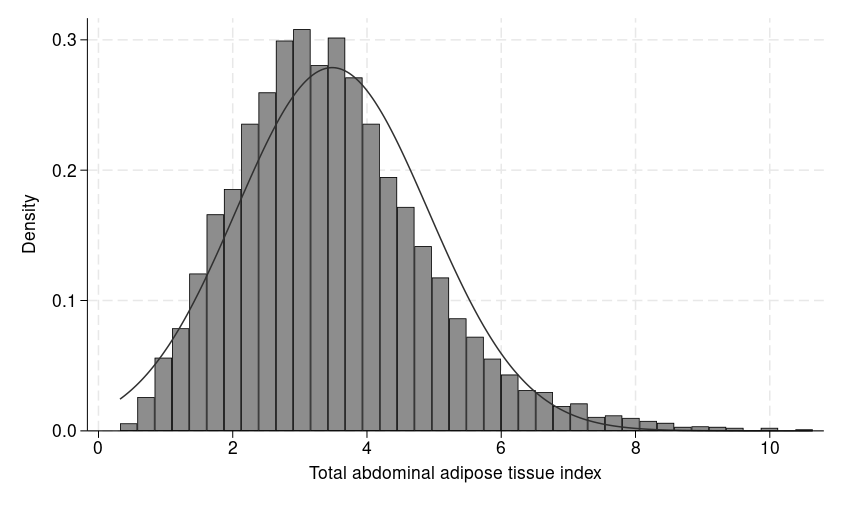

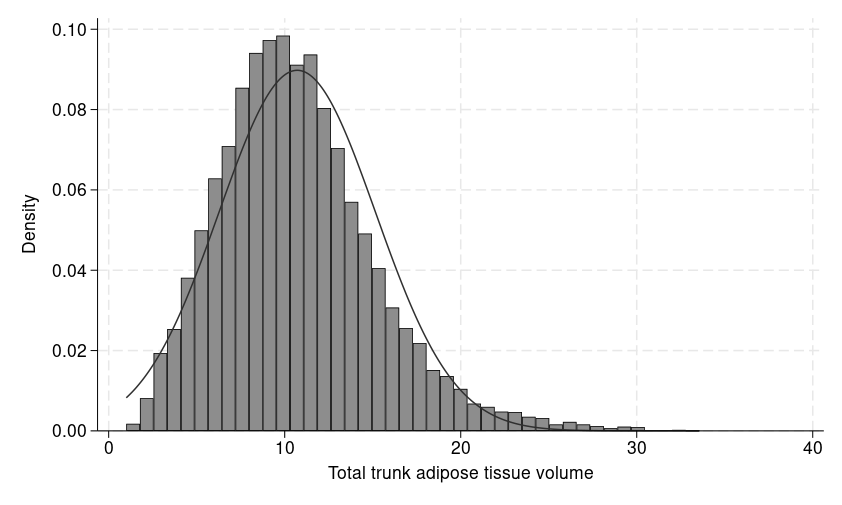

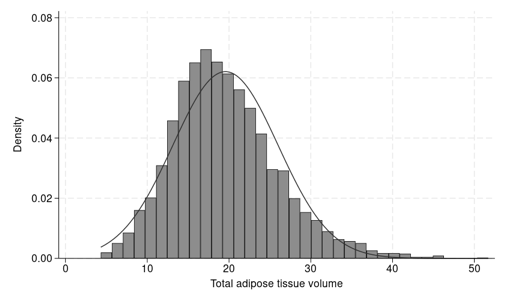

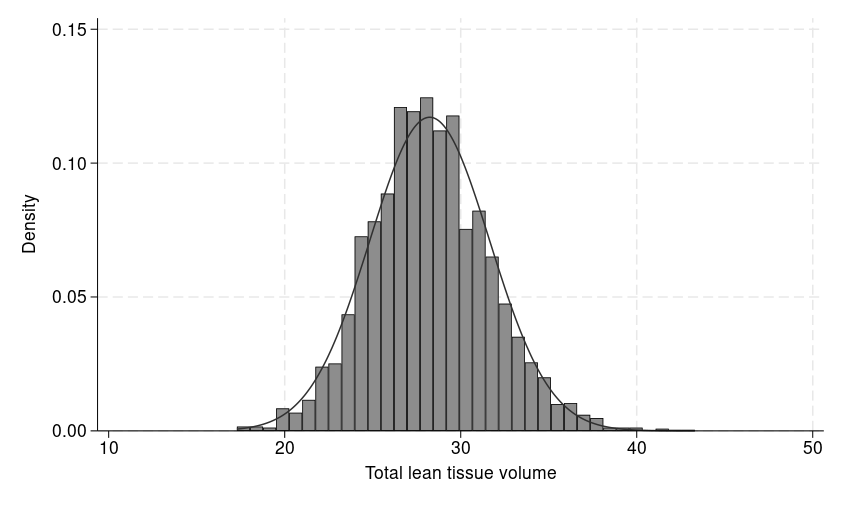

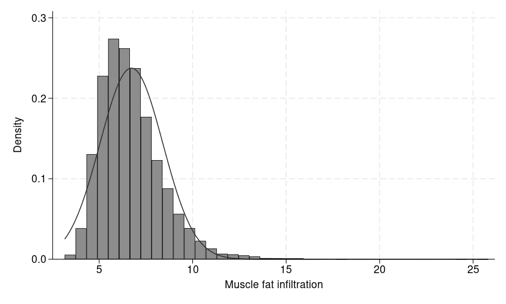

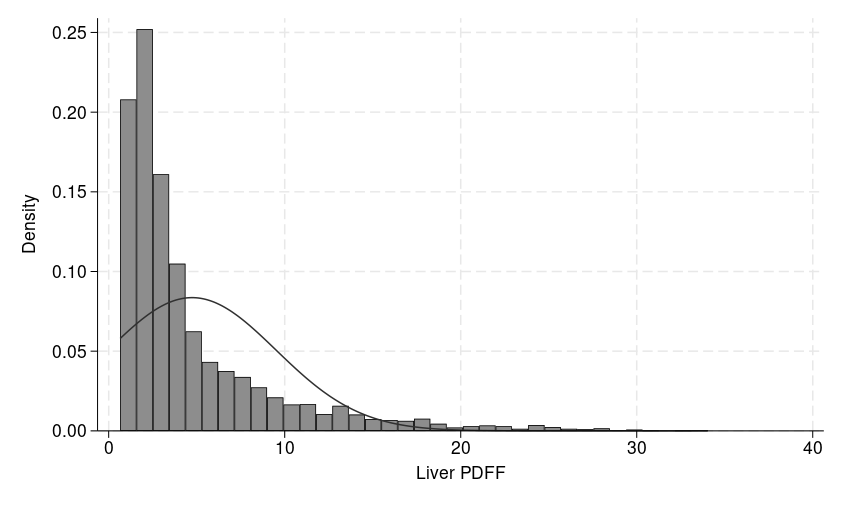

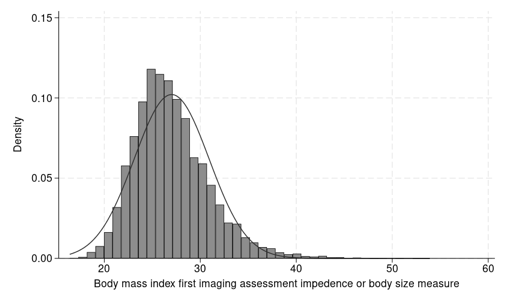

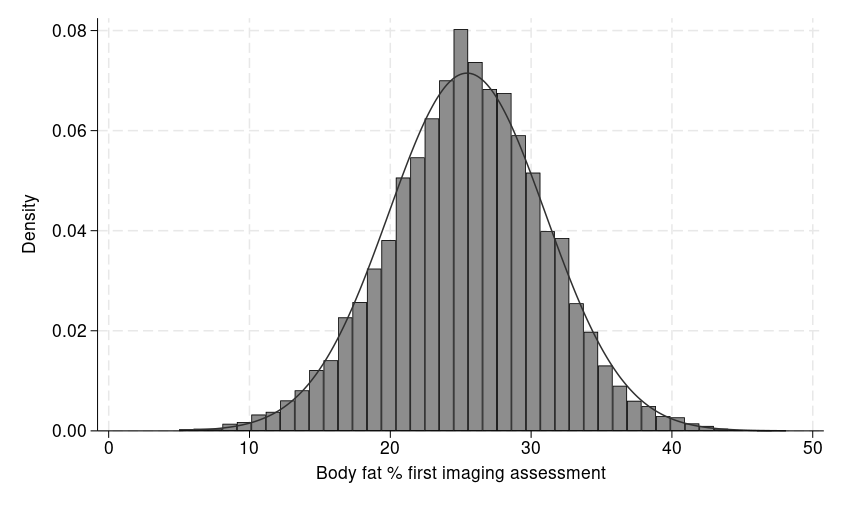

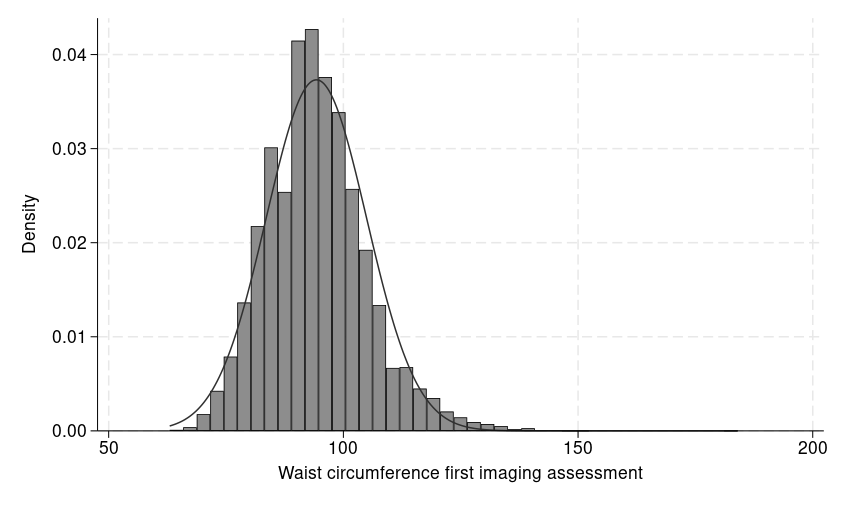

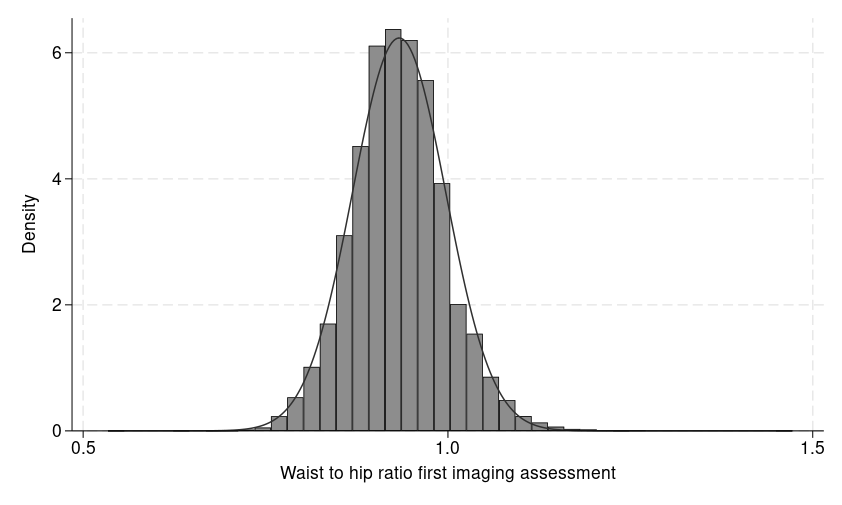


A)

B)

C)

D)

E)

F)

G)

H)

I)

J)

K)

L)

M)

N)

O)

P)

## Supplementary Figure 3. Minimally adjusted models of body composition measures in relation to (A) IGF-I and (B) SHBG geometric mean concentrations (n=16,237).

Models were adjusted for age and ethnicity. MRI measures are found in the top portion of the figure, with classic anthropometric in the lower portion. The relative geometric mean is the ratio of log-transformed values relative to the first quintile. P-value for heterogeneity assesses evidence of an overall assocation; p-value for non-linearity assesses departure from linearity per incremental increase of exposure (significance indicates non-linearity).

Abbreviations: CI, confidence interval; MRI, magnetic resonance imaging; N, number of participants; nmol/L, nanomoles per litre; PDFF, proton density fat fraction; Q, quintile.

## Supplementary Figure 4. Minimally adjusted models of body composition measures in relation to (A) total testosterone and (B) free testosterone geometric mean concentrations (n=16,237).

Models were adjusted for age and ethnicity. MRI measures are found in the top portion of the figure, with classic anthropometric in the lower portion. The relative geometric mean is the ratio of log-transformed values relative to the first quintile. P-value for heterogeneity assesses evidence of an overall assocation; p-value for non-linearity assesses departure from linearity per incremental increase of exposure (significance indicates non-linearity).

Abbreviations: CI, confidence interval; MRI, magnetic resonance imaging; N, number of participants; nmol/L, nanomoles per litre; PDFF, proton density fat fraction; pmol/L, picomoles per litre; Q, quintile.

## Supplementary Figure 5. Multivariable adjusted models comparing body composition measures to the mean of the baseline and repeated biomarker measurements for (A) IGF-I and (B) SHBG concentrations (n=2,681).

MRI measures are found in the top portion of the figure, with classic anthropometric in the lower portion. The relative geometric mean is the ratio of log-transformed values relative to the first quintile. P-value for heterogeneity assesses evidence of an overall assocation; p-value for non-linearity assesses departure from linearity per incremental increase of exposure (significance indicates non-linearity).

Abbreviations: CI, confidence interval; MRI, magnetic resonance imaging; N, number of participants; nmol/L, nanomoles per litre; PDFF, proton density fat fraction; Q, quintile.

Models adjusted for: age, ethnicity, education, Townsend Deprivation Index, region, employment, cohabitation status, height, diabetes, cigarette smoking, alcohol consumption, and physical activity.

## Supplementary Figure 6. Multivariable adjusted models comparing body composition measures using the mean of the baseline and repeated biomarker measurements for (A) total testosterone and (B) free testosterone (n=2,681).

**[nmol/L]**

MRI measures are found in the top portion of the figure, with classic anthropometric in the lower portion. The relative geometric mean is the ratio of log-transformed values relative to the first quintile. P-value for heterogeneity assesses evidence of an overall assocation; p-value for non-linearity assesses departure from linearity per incremental increase of exposure (significance indicates non-linearity).

Abbreviations: CI, confidence interval; MRI, magnetic resonance imaging; N, number of participants; PDFF, proton density fat fraction; pmol/L, picomoles per litre; Q, quintile.

Models adjusted for: age, ethnicity, education, Townsend Deprivation Index, region, employment, cohabitation status, height, diabetes, cigarette smoking, alcohol consumption, and physical activity.

## Supplementary Figure 7. Multivariable adjusted models comparing body composition measures to (A) IGF-I and (B) SHBG concentrations restricted to participants where BMI changed less than 5% between recruitment and imaging visits (n=10,408).

MRI measures are found in the top portion of the figure, with classic anthropometric in the lower portion. The relative geometric mean is the ratio of log-transformed values relative to the first quintile. P-value for heterogeneity assesses evidence of an overall assocation; p-value for non-linearity assesses departure from linearity per incremental increase of exposure (significance indicates non-linearity).

Abbreviations: CI, confidence interval; MRI, magnetic resonance imaging; N, number of participants; nmol/L, nanomoles per litre; PDFF, proton density fat fraction; Q, quintile.

Models adjusted for: age, ethnicity, education, Townsend Deprivation Index, region, employment, cohabitation status, height, diabetes, cigarette smoking, alcohol consumption, and physical activity.

## Supplementary Figure 8. Multivariable adjusted models comparing body composition measures to (A) total testosterone and (B) free testosterone concentrations restricted to participants where BMI changed less than 5% between recruitment and imaging visits (n=10,408).

**[nmol/L]**

MRI measures are found in the top portion of the figure, with classic anthropometric in the lower portion. The relative geometric mean is the ratio of log-transformed values relative to the first quintile. P-value for heterogeneity assesses evidence of an overall assocation; p-value for non-linearity assesses departure from linearity per incremental increase of exposure (significance indicates non-linearity)

Abbreviations: CI, confidence interval; MRI, magnetic resonance imaging; N, number of participants; nmol/L, nanomoles per litre; PDFF, proton density fat fraction; pmol/L, picomoles per litre; Q, quintile.

Models adjusted for: age, ethnicity, education, Townsend Deprivation Index, region, employment, cohabitation status, height, diabetes, cigarette smoking, alcohol consumption, and physical activity.
